# Data on novel C fibers@MoSe_2_ nanoplates core–shell composite for highly efficient solar-driven photocatalytically degrading environmental pollutants

**DOI:** 10.1016/j.dib.2018.01.103

**Published:** 2018-02-08

**Authors:** Meng Wang, Zhijian Peng, Jingwen Qian, Hong Li, Zengying Zhao, Xiuli Fu

**Affiliations:** a School of Engineering and Technology, China University of Geosciences, Beijing 100083, PR China; b School of Science, China University of Geosciences, Beijing 100083, PR China; c State Key Laboratory of Information Photonics and Optical Communications, and School of Science, Beijing University of Posts and Telecommunications, Beijing 100876, PR China

**Keywords:** Environmental pollutants, Solar-driven photocatalytic degradation, MoSe_2_ nanoplates, Carbon fiber, Composite

## Abstract

The data presented in this article are related to a research article entitled ‘Highly efficient solar-driven photocatalytic degradation on environmental pollutants over a novel C fibers@MoSe_2_ nanoplates core–shell composite’ (Wang et al., 2018) [1]. In this article, we report original data on the synthesis processes optimization of the proposed composite together with its formation mechanism. The report includes the composition, microstructure and morphology of the corresponding samples, and the photocatalytic activity and stability of the optimal composite. Compared with commercially available MoSe_2_ powder, the reaction rate constant of the optimal composite catalyst for the degradation of methylene blue (MB) and rhodamine B (RhB) under simulated sunlight irradiation (SSI) could be increased in a factor of about 14 and 8, respectively. The data are presented in this format to allow the comparison with those from other researchers in this field, and understanding the synthesis and photocatalysis mechanism of similar catalysts.

**Specifications table**

**Table:** 

Subject area	*Environmental engineering, Environmental science, Chemical engineering, Materials science, Materials engineering*
More specific subject area	*Photocatalytic degradation, New energy devices*
Type of data	*Tables, Figures*
How data was acquired	*X-ray diffraction (XRD, Rigaku D/max-RB, Japan), Field emission scanning electron microscope (FE-SEM, Quanta FEG-650, America), Photocatalytic reaction system (PCX50A Discover, Beijing Perfectlight Technology Co., Beijing, China)*
Data format	*Raw and analyzed data*
Experimental factors	*The amounts of the used reaction resources: absolute ethanol (constantly 5 mL), MoO*_*3*_*powder (1.0–1.6 g), Se powder (0.5–3.0 g), pre-oxidized polyacrylonitrile (PAN) fiber (constantly 0.15 g).Temperature: 900–1100 °C for synthesizing the photocatalystsReaction time: 1 h for synthesizing the photocatalysts*
Experimental features	*The designed experiments included the optimization of synthesis processes and comparison on the photocatalytic degradation of MB, RhB, p-chlorophenol (4-CP) and K*_*2*_*Cr*_*2*_*O*_*7*_*(Cr, VI)*
Data source location	*The composite was grown in Beijing, China*
Data accessibility	*The data are available with this article*

**Value of the data**

• The data on the synthesis processes optimization of the C fibers@MoSe_2_ nanoplates core–shell composite (NPCSC) could give an insight into its formation and photocatalysis mechanisms to other researchers interested in the synthesis and application of photocatalysts.
• The data can be used by researchers interested in developing other composite photocatalysts and understanding their photocatalytic mechanism.
• The data can be used by researchers interested in developing new energy materials, and energy storage and conversion devices.

## 1 Data

The data presented in this paper are related to a research article entitled ‘Highly efficient solar-driven photocatalytic degradation on environmental pollutants over a novel C fibers@MoSe_2_ nanoplates core–shell composite’ [1].

**Fig. 1:**
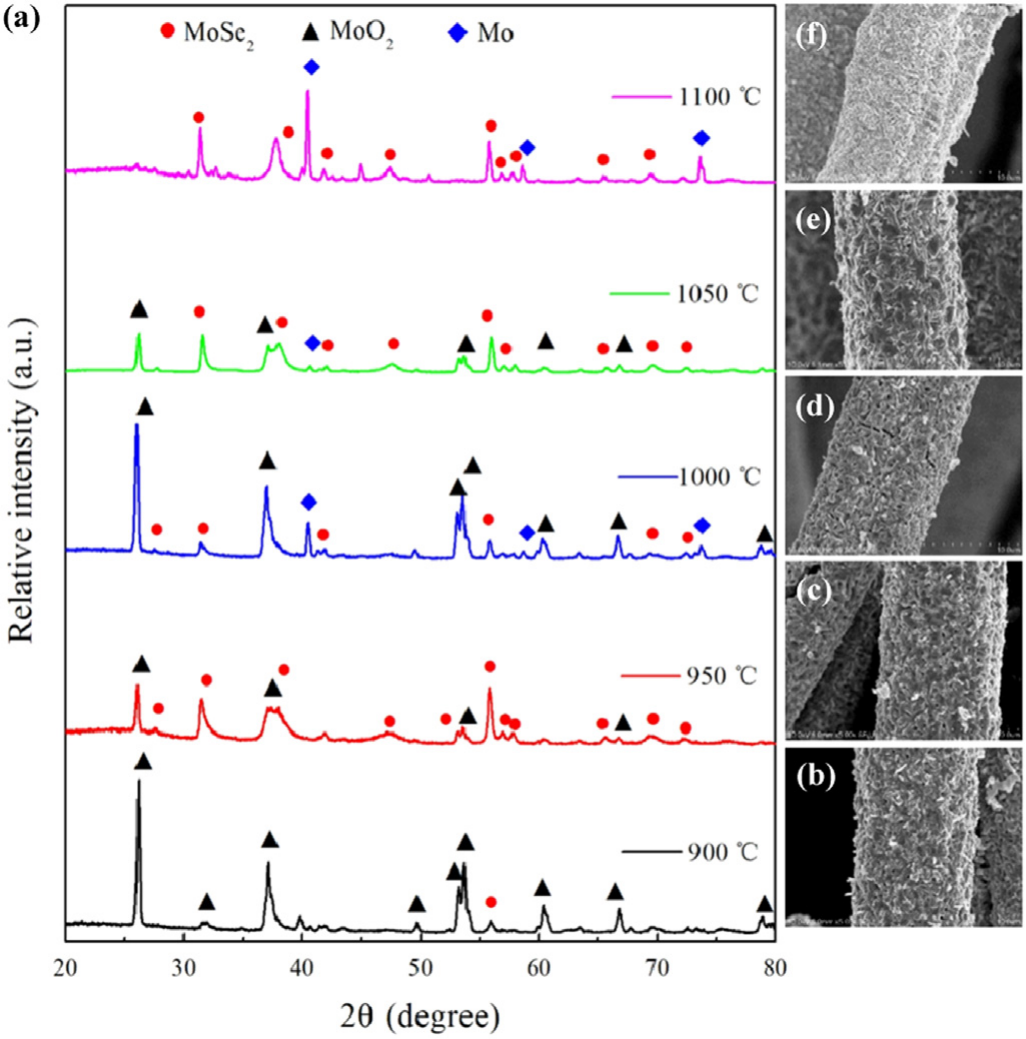
(a) Typical XRD patterns of the samples prepared at different temperatures, and their corresponding SEM images: (b) 900, (c) 950, (d) 1000, (e) 1050 and (f) 1100 °C. In this group of experiments, 2.0 g of MoO_3_ powder and 0.5 g of Se powder were used.

**Fig. 2:**
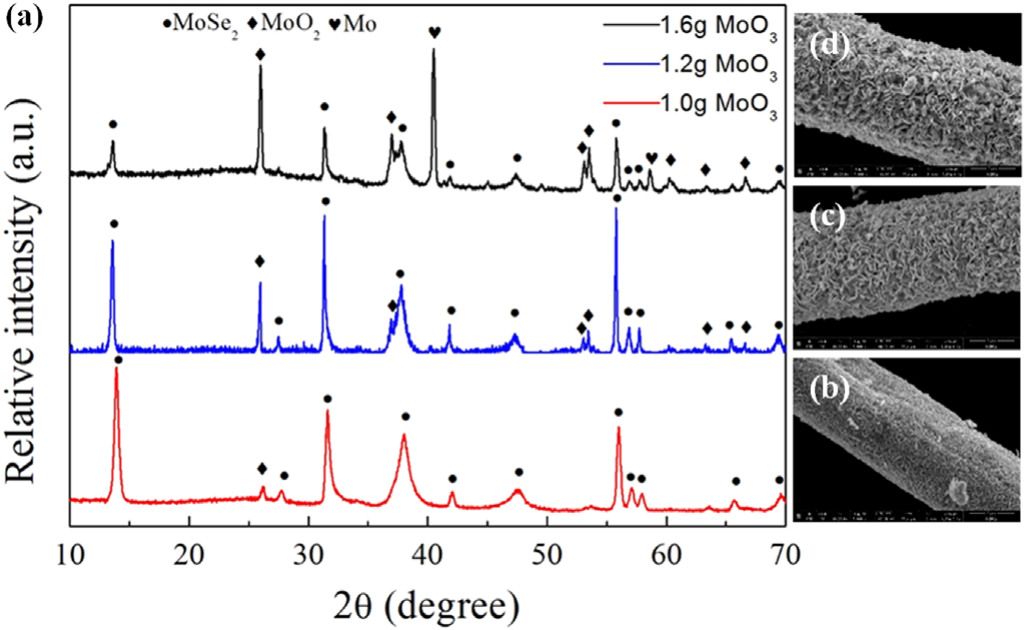
(a) Typical XRD patterns of the samples prepared at a constant temperature of 1100 °C with different amounts of MoO_3_ and a fixed amount (2.0 g) of Se powder, and their corresponding SEM images with (b) 1.0, (c) 1.2 and (d) 1.6 g of MoO_3_ powder.

**Fig. 3:**
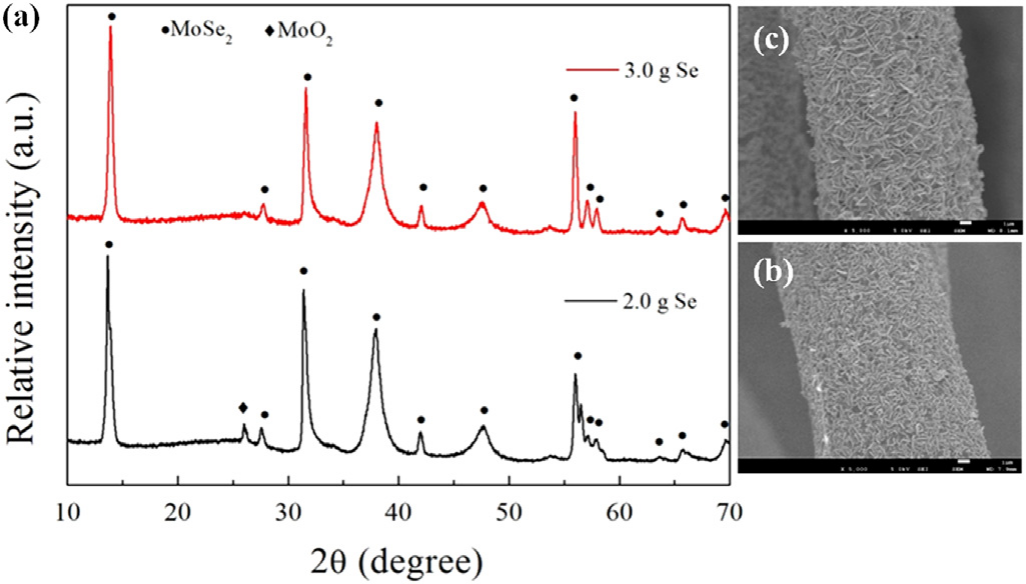
(a) Typical XRD patterns of the samples prepared with different amounts of Se powder but a fixed amount (1.0 g) of MoO_3_ powder at a constant temperature of 1100 °C, and their corresponding SEM images with different amounts of Se powder: (b) 2.0 and (c) 3.0 g.

**Fig. 4:**
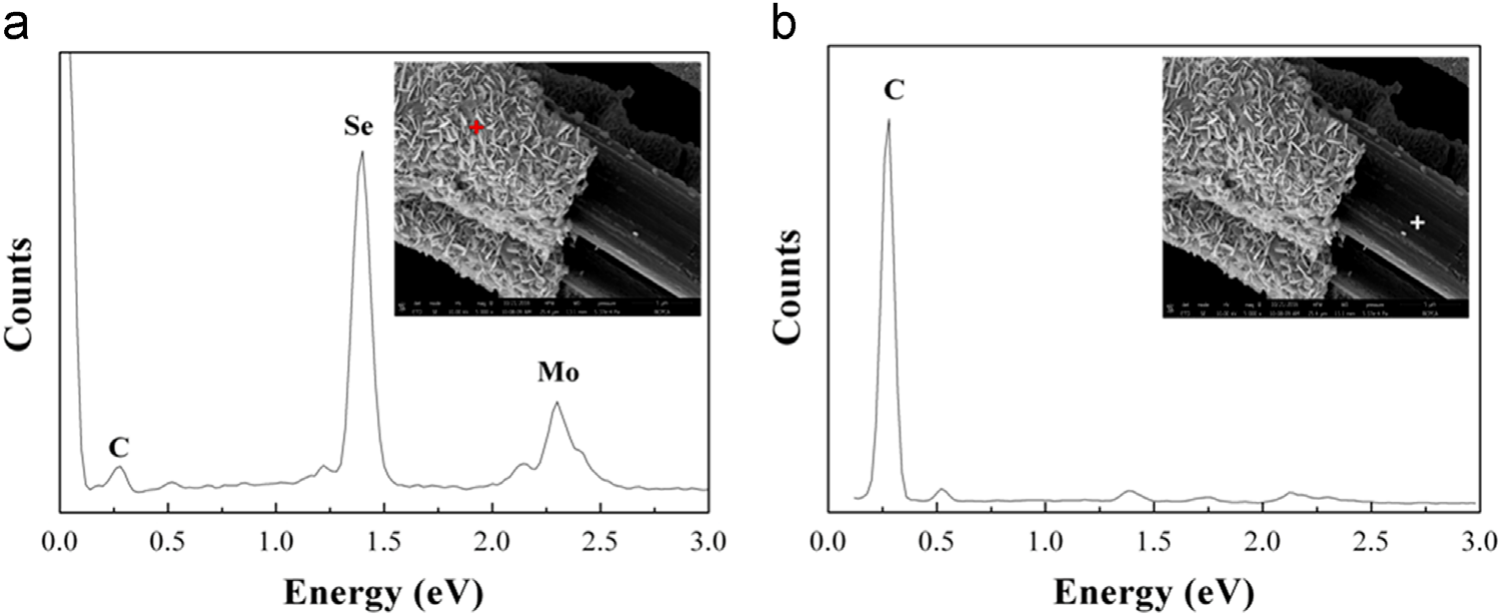
EDX spectra on the outer shell (a) and inner core (b) of typical fractured fiber after ultrasonic oscillation in ethyl alcohol (see the insets).

**Fig. 5:**
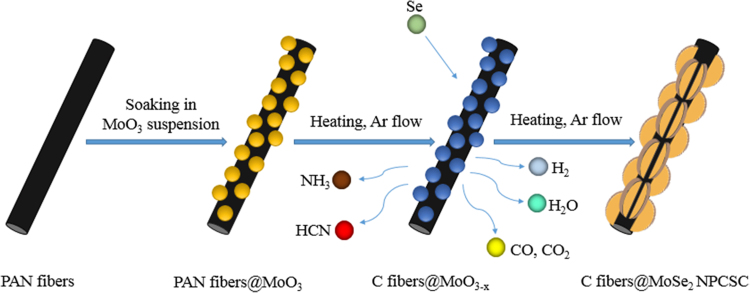
Formation mechanism of the C fibers@MoSe_2_ NPCSC.

**Fig. 6:**
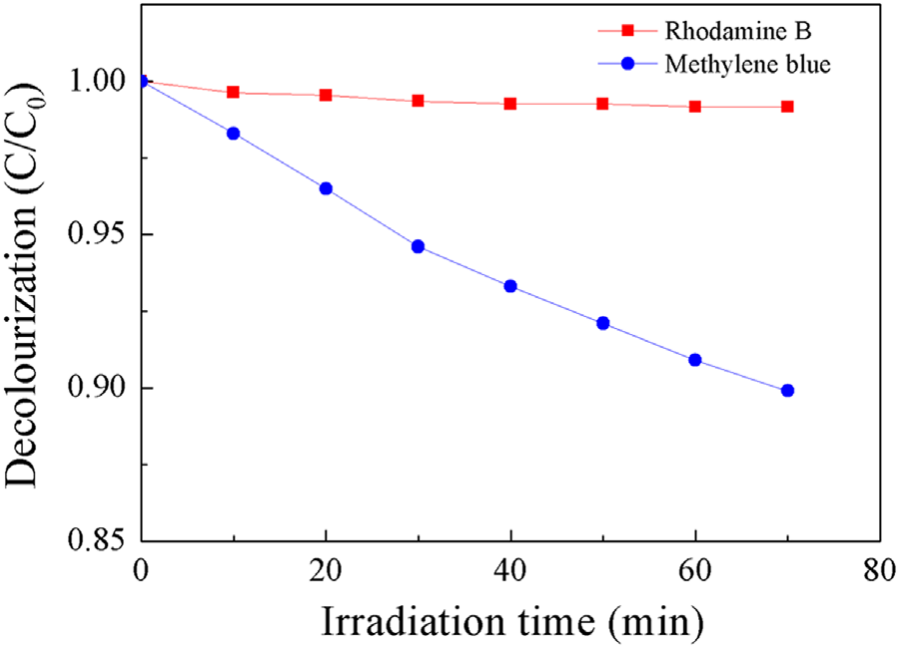
The decolourization of organic dyes MB and RhB during photodegradation under SSI without any catalyst.

**Fig. 7:**
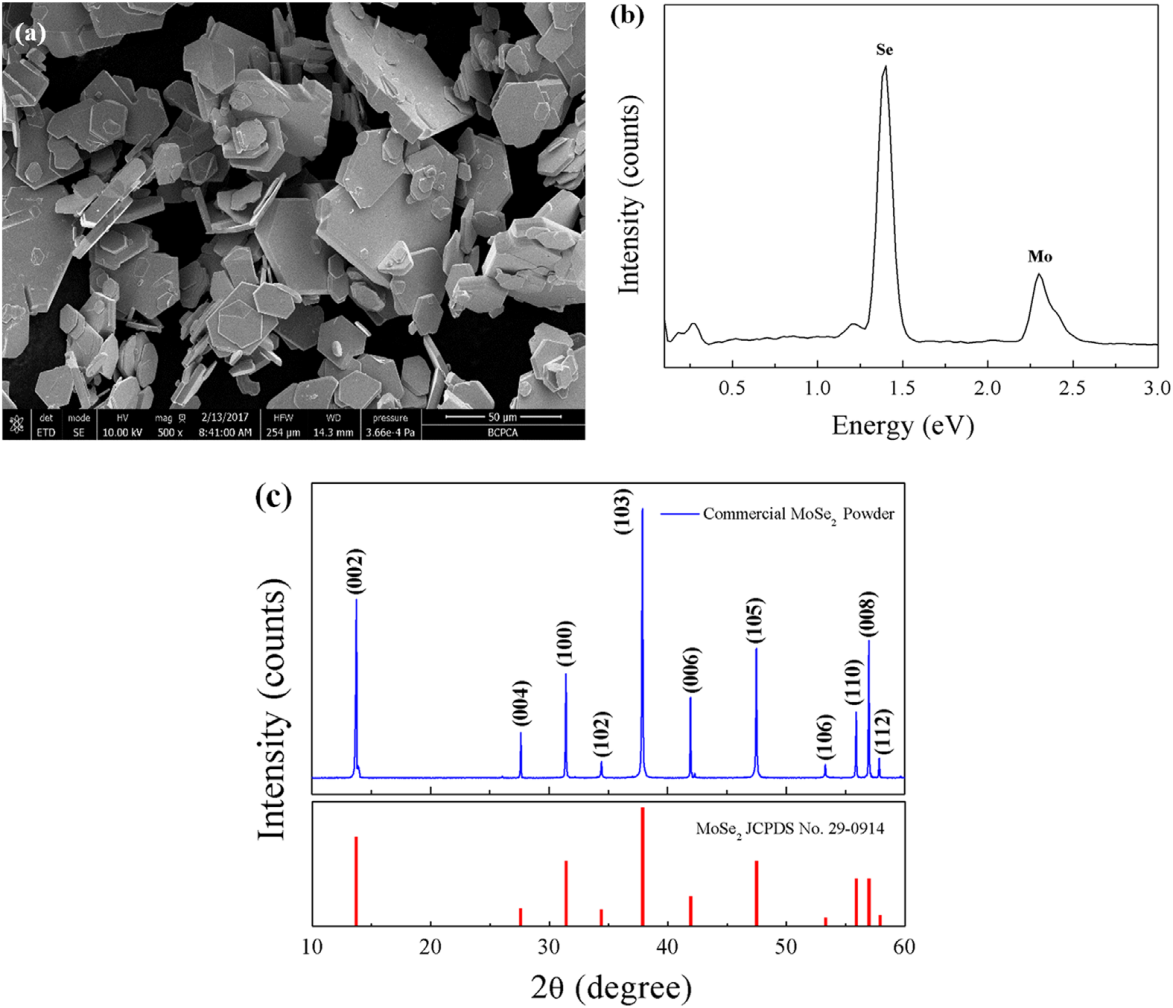
Composition and microstructure of the commercially available MoSe_2_ powder. (a) Typical SEM image, indicating that the powder consists completely of MoSe_2_ nanoplates. (b) Typical EDX spectrum on the imaging area of (a), revealing that the powder is composed of only Mo and Se. (c) Typical XRD pattern of the sample, in which the diffraction peaks are matching well with those of MoSe_2_ phase (JCPDS card no. 29-0914). All these results reveal that the commercially available powder is composed of pure MoSe_2_ nanoplates.

**Fig. 8:**
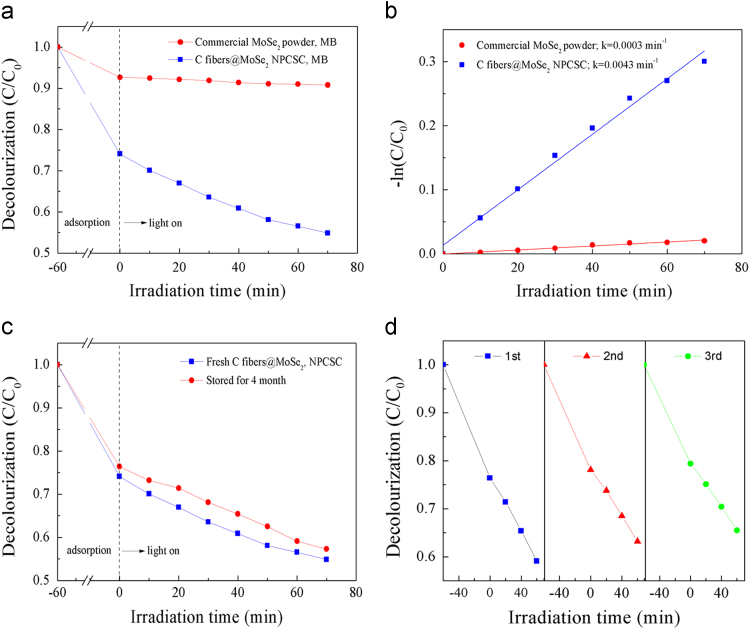
Photocatalytic degradation on MB over the sample. (a) Decolourization effects on MB under SSI over the as-prepared C fibers@MoSe_2_ NPCSC and commercially available MoSe_2_ powder, and (b) their corresponding *-ln(C/C*_*0*_*) vs. irradiation time* plots. (c) Decolourization effects on MB under SSI over fresh C fibers@MoSe_2_ NPCSC and that stored for 4 months. (d) Recycle experiment of photocatalytic degradation on MB under SSI over the as-prepared C fibers@MoSe_2_ NPCSC.

**Fig. 9:**
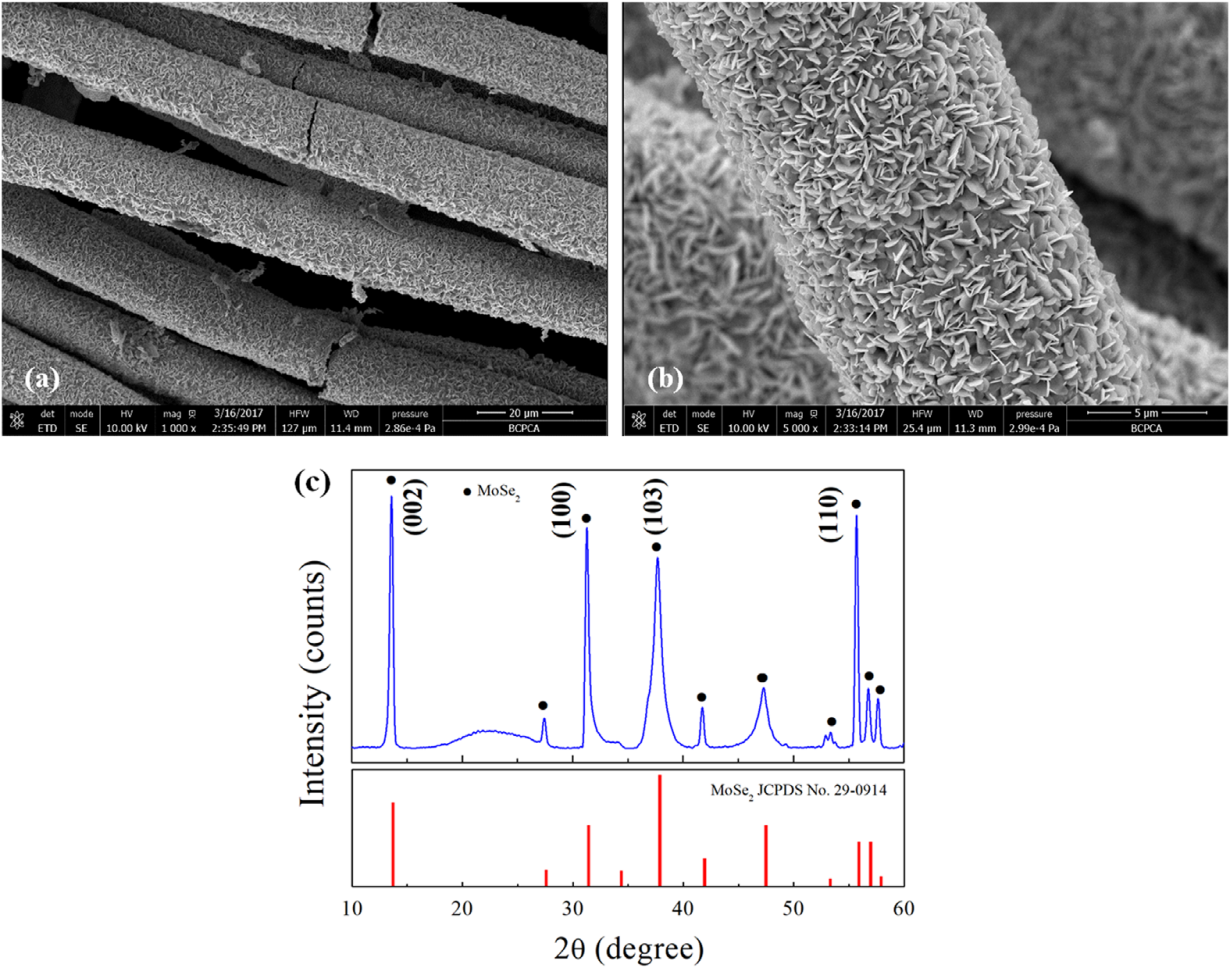
Typical low- and high- magnification SEM image (a, b) with the corresponding XRD pattern (c) of the C fibers@MoSe_2_ NPCSC catalyst after being applied in photodegrading MB under SSI for 70 min.

**Fig. 10:**
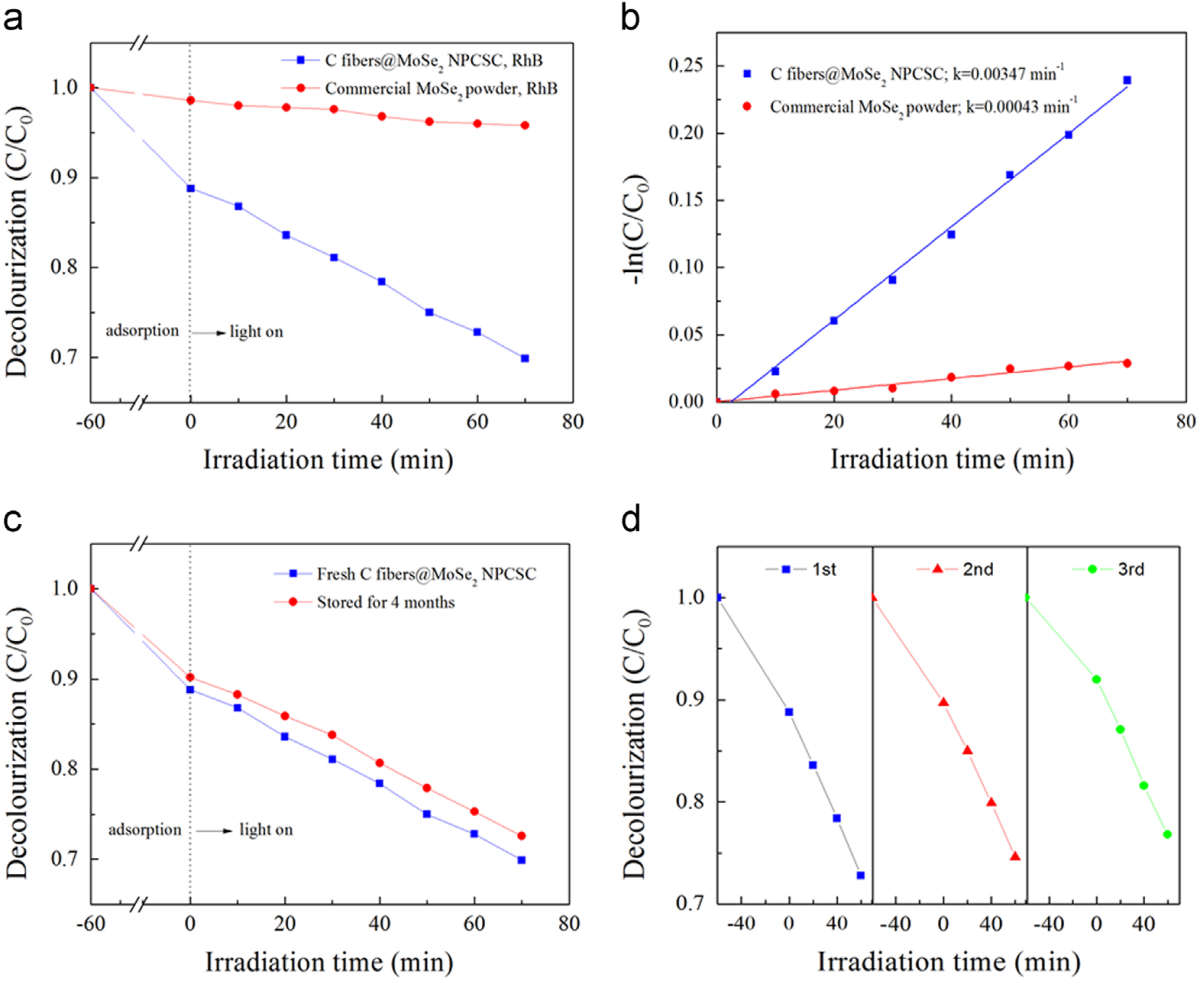
Photocatalytic degradation on RhB over the sample. (a) Decolourization effects on RhB under SSI over the as-prepared C fibers@MoSe_2_ NPCSC and commercially available MoSe_2_ powder, and (b) their corresponding *-ln(C/C*_*0*_*) vs. irradiation time* plots. (c) Decolourization effects on RhB under SSI over fresh C fibers@MoSe_2_ NPCSC and that stored for 4 months. (d) Recycle experiment of photocatalytic degradation on RhB under SSI over the as-prepared C fibers@MoSe_2_ NPCSC.

**Fig. 11:**
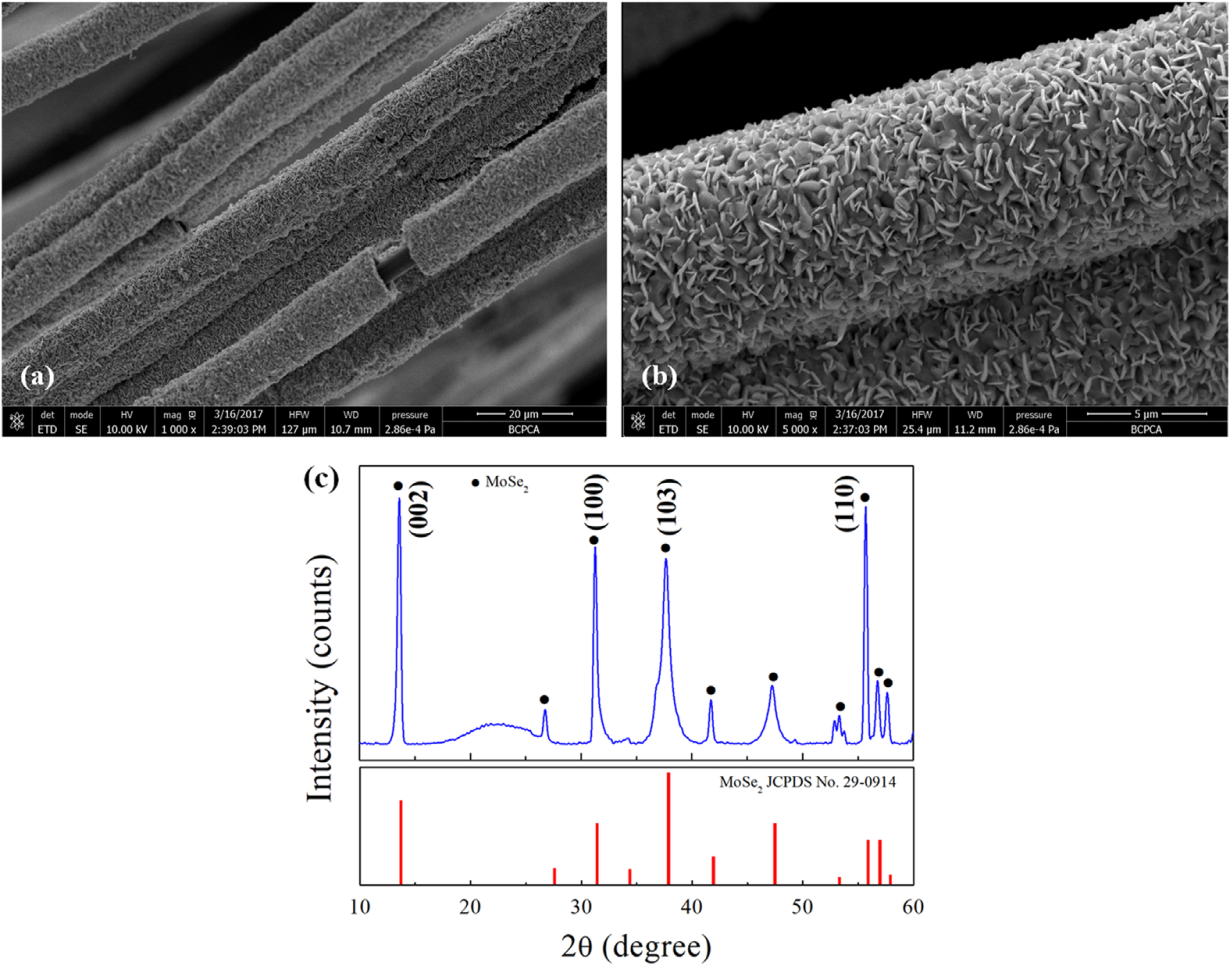
Typical low- and high- magnification SEM image (a, b) with the corresponding XRD pattern (c) of the C fibers@MoSe_2_ NPCSC catalyst after being applied in the photodegradation of RhB under SSI for 70 min.

**Fig. 12:**
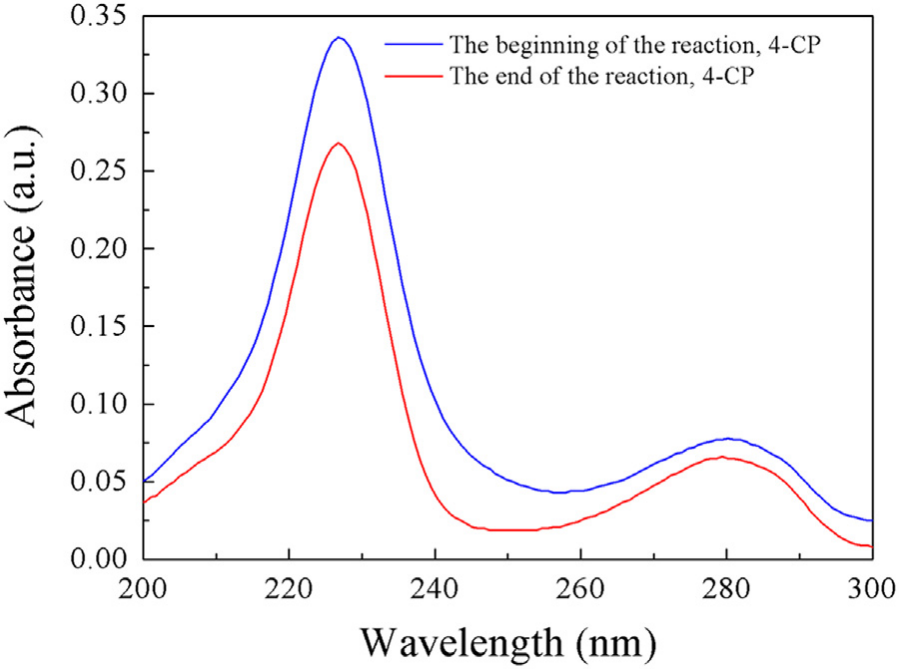
UV–vis absorption spectra of 4-CP at the beginning (blue line) and end (red line) of the photocatalytic reaction. As can be seen from Fig. 12, after a long period of SSI, the intensity of UV absorption peak of 4-CP (225 nm and 280 nm) decreased significantly. This result illustrates that the applied 4-CP has been substantially photocatalytically degraded.

**Fig. 13:**
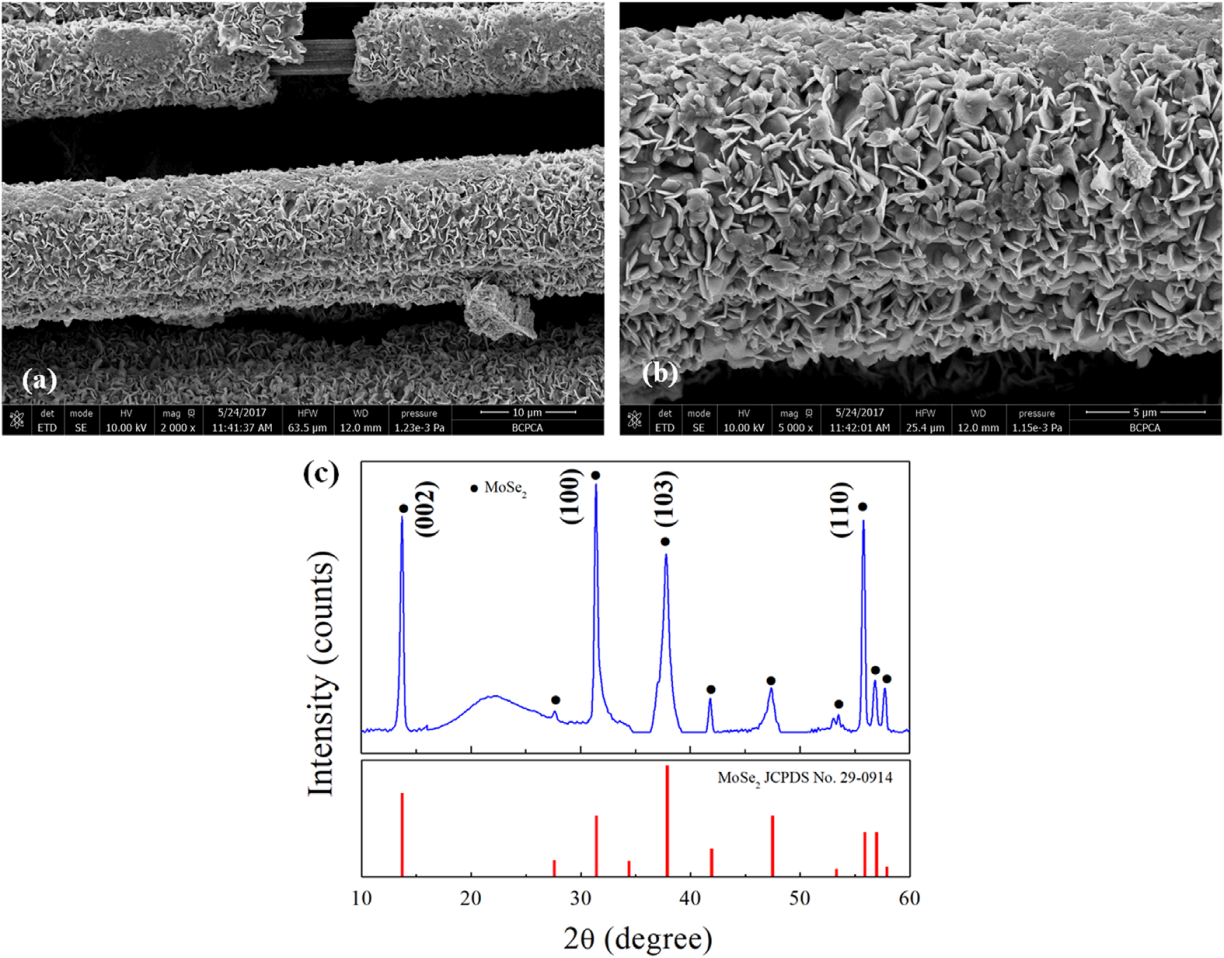
Typical low- and high- magnification SEM images (a, b) and the corresponding XRD pattern (c) of the C fibers@MoSe_2_ NPCSC catalyst after being applied in the photodegradation of 4-CP solutions under SSI for 70 min.

**Fig. 14:**
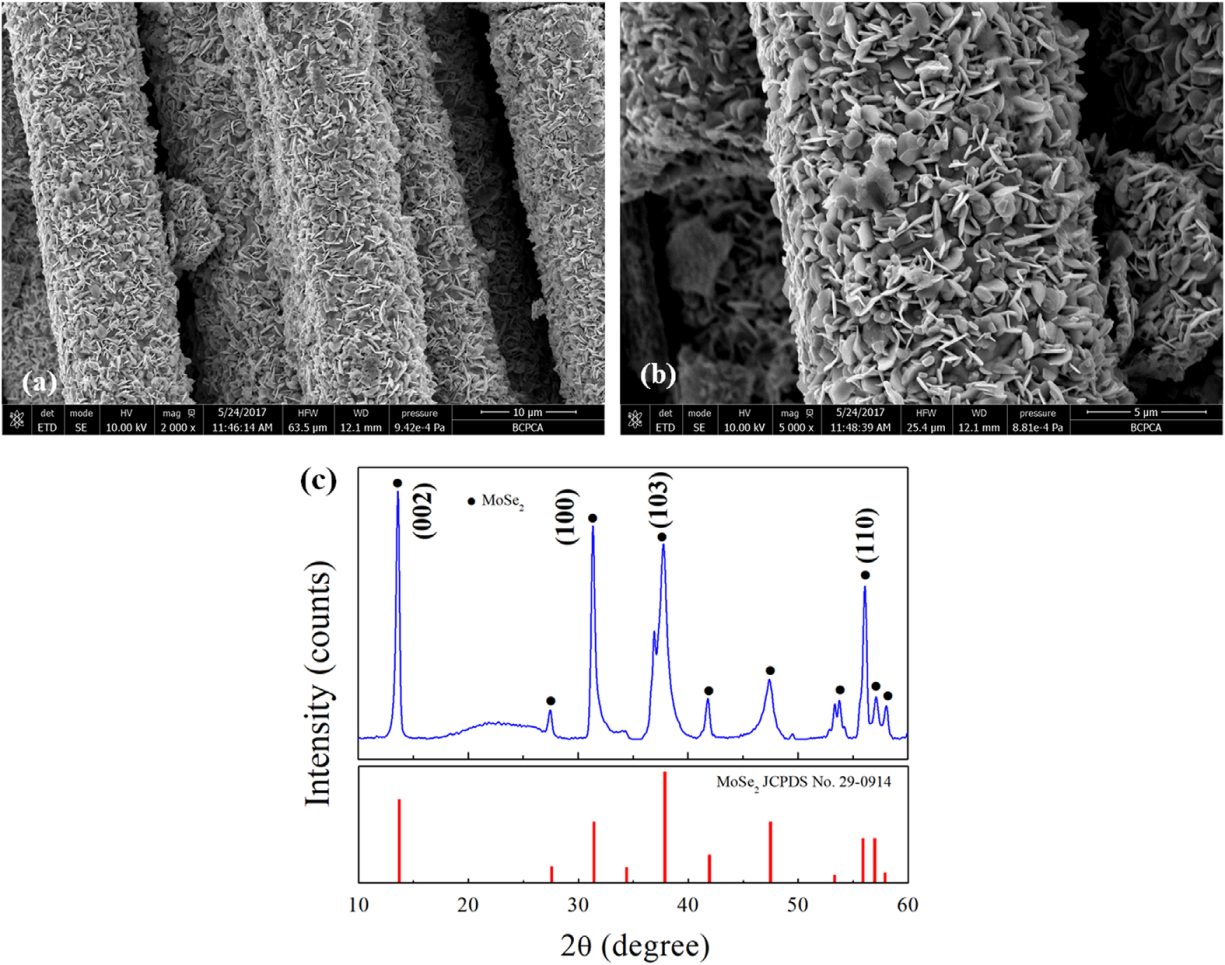
Typical low- and high- magnification SEM images (a, b) and the corresponding XRD pattern (c) of the C fibers@MoSe_2_ NPCSC catalyst after being applied in the photodegradation of K_2_Cr_2_O_7_ solution under SSI for 120 min.

It includes data on the synthesis processes optimization and formation mechanism of the present C fibers@MoSe_2_ NPCSC (Fig. 1, Fig. 2, Fig. 3, Fig. 4, Fig. 5), which reveal that numerous MoSe_2_ thin nanoplates are grown in-situ, densely and even vertically on the surface of the C fibers, forming the optimal core–shell composite. Data on the photocatalytic performance and stability of the optimal composite catalyst are also presented (Fig. 6, Fig. 7, Fig. 8, Fig. 9, Fig. 10, Fig. 11, Fig. 12, Fig. 13, Fig. 14). In addition, data on the activity for the photodegradation of 4-CP and Cr (VI) over the present C fibers@MoSe_2_ NPCSC and other photocatalysts are compared in Table 1, Table 2.

**Table 1:** Comparison on the photocatalytic degradation of 4-CP over the C fibers@MoSe_2_ NPCSC with those over TiO_2_-based catalysts reported in literature.

**Catalysts**	**Light source**	**Light intensity (mW/cm**^**2**^**)**	**Catalyst concentration (g/L)**	**Stirring**	**Decolourization rate**	**k (min**^**−1**^**)**	**Recycling times**	**Refs.**
C fiber@MoSe_2_ NPCSC	5 W LED lamp	36	1	no	19.4% in 70 min	0.00289	3	[1]
MoS_2_ and WS_2_ nanocluster sensitized TiO_2_ nanoparticles	300 W tungsten halogen lamp *λ* ≥ 400 nm	–	1	no	63% in 300 min	–	–	[5]
C-modified TiO_2_ nanoparticles	250 W Xe lamp *λ*≥ 420 nm	30	1	yes	77.5% in 240 min	0.0061	–	[6]
N-F-codoped TiO_2_ nanoparticles	500 W Xe lamp *λ*≥ 400 nm	–	2	yes	72.48% in 300 min	–	5	[7]
N-doped TiO_2_ nanoparticles	500 W Xe lamp *λ*≥ 400 nm	–	0.5	yes	63.5% in 300 min	–	–	[8]

**Table 2:** Comparison on the photocatalytic reduction of Cr (VI) over the present C fibers@MoSe_2_ NPCSC catalyst with those over other semiconductor-based catalysts reported in literature.

**Catalysts**	**Light source**	**Light intensity (mW/cm**^**2**^**)**	**Catalyst concentration (g/L)**	**Stirring**	**Decolourization rate**	**k (min**^**-1**^**)**	**Recycling times**	**Refs.**
C fiber@MoSe_2_ NPCSC	5 W LED lamp	36	1	no	34.7% in 120 min	0.0034	3	[1]
C quantum dots decorated MoSe_2_	300 W Xe lamp *λ*≥ 400 nm	741	1	yes	99% in 180 min	0.026	3	[9]
Hexagonal 2H-MoSe_2_ nanoparticles	300 W Xe lamp *λ*≥ 400 nm	741	1	yes	94% in 180 min	0.027	3	[10]
MoSe_2_ nanosheets/TiO_2_ nanoparticles composite	400 W metal halogen lamp *λ*≥ 400 nm	–	1	yes	91% in 120 min	0.0141	–	[11]
MoSe_2_ nanoparticles	400 W metal halogen lamp *λ*≥ 400 nm	–	1	yes	95% in 250 min	–	5	[12]
3D MoS_2_/r-GO aerogel	300 W Xe lamp	545	0.67	yes	92% in 120 min	–	–	[13]
2D MoS_2_ nanosheet coated Bi_2_S_3_ discoids	300 W Xe lamp *λ*≥ 400 nm	700	0.25	yes	97% in 30 min	–	3	[14]

## 2 Experimental design, materials and methods

Novel highly efficient C fibers@MoSe_2_ NPCSC photocatalyst for environmental remediation was described by Wang et al. [1]. In order to improve the photocatalytic performance of the composite, the synthesis processes were optimized by changing the reaction temperature from 900 to 1100 °C, and adjusting the applied amounts of MoO_3_ powder from 1.0 to 1.6 g in 5 mL absolute ethanol and Se powder from 0.5 to 3.0 g. All the prepared samples were characterized by XRD and SEM. And all the experiments were conducted in duplicates.

From Fig. 1, it can be seen that the sample prepared at 900 °C almost consists of pure MoO_2_ nanoparticles. As the temperature increased, MoO_2_ nanoparticles were further, gradually reduced into MoSe_2_, resulting in nanoplates on the surfaces of the carbon fibers. When it increased up to 1100 °C, MoSe_2_ nanoplates had completely replaced MoO_2_ nanoparticles, although there was still a little amount of metallic Mo in the sample.

This figure reveals that when 1.0 g of MoO_3_ powder was used, the desirable product had MoSe_2_ nanoplates in a quite high density, and the by-products such as MoO_2_ were in the least amount.

Fig. 3 reveals that, under the present condition, when the applied amount of Se powder increased up to 3.0 g, the sample had been a pure C fibers@MoSe_2_ NPCSC.

The recorded EDX spectrum on the outer shell indicates that it consists of mainly Mo and Se atoms with very little of C atoms (see Fig. 4a). In combination with its morphology, it can be seen that the outer shell is composed of molybdenum selenide nanoplates. In addition, a small amount of C atoms was also detected on the molybdenum selenide nanoplates. The EDX spectrum on the inner core reveals that it is composed of only C atoms (see Fig. 4b), implying that the inner core is of elemental carbon.

Based on the results presented in Fig. 1, Fig. 2 in Ref. [1] and the corresponding discussion, in combination with the present Fig. 1, Fig. 2, Fig. 3, Fig. 4, a possible formation mechanism called in-situ “symplastic growth” can be used to explain the growth of the present C fibers@MoSe_2_ NPCSC. The whole process can be schematically shown in Fig. 5. In the first step, a composite of PAN fibers@MoO_3_ particles was formed by soaking the PAN fibers in MoO_3_ suspension, where the MoO_3_ particles were uniformly coating on the surface of the PAN fibers. In the second step, at 400–600 °C under the action of inert gas, the oxygen-containing functional groups of the pre-oxidized PAN fibers were dehydrated and cross-linked to form a more stable trapezoidal structure. The trapezoid molecules were connected into a graphene-like structure by the dehydrogenation reaction. When the temperature raised up to above 600 °C, in the third step, denitrification reaction would occur, forming structured C fibers and releasing H_2_, NH_3_, HCN, H_2_O and so on [2]. Synchronously, the partially pyrolyzed C reacted with MoO_3_ to produce MoO_2_ and reducing gas CO. As the amounts of reducing H_2_ and CO gases increased, and more Se vapor was fed from the upstream, MoO_2_ nanocrystals were further selenized to form MoSe_2_ nanoplates, finally producing the C fibers@MoSe_2_ NPCSC.

Fig. 6 reveals that without photocatalysts, MB is self-sensitized but RhB is stable under SSI. Under the present conditions, MB will be decolourized by SSI at about 10%, but without photocatalysts, no photodegradation under SSI could be observed on RhB.

Fig. 8 shows the decolourization effects on MB under SSI over the as-prepared C fibers@MoSe_2_ NPCSC and commercially available MoSe_2_ powder, respectively. As is seen from Fig. 8a, a dark adsorption for 60 min was performed prior to light irradiation so as to reach the adsorption–desorption equilibrium. In this stage, the decolourized MB over the C fibers@MoSe_2_ NPCSC was 25.9%, while that over the MoSe_2_ powder was only 7.3%. During the photocatalytic degradation, the degraded MB over the C fibers@MoSe_2_ NPCSC reached 19.2%, whereas that over the commercially bought MoSe_2_ powder was only 1.9%. This result reveals that the commercially bought MoSe_2_ powder has no usable photocatalytic activity on the degradation of MB; however, after compositing with C fiber, the photocatalytic performance of MoSe_2_ nanoplates can be greatly enhanced.

The photocatalytic degradation of MB follows the pseudo-first-order kinetics as described by the equation of −ln(*C*/*C*_0_) =*kt* [3], [4]. Through this equation, straight lines can be fitted into Fig. 8b, in which the slope of the straight lines can be explained as the photocatalytic reaction rate constant *k*. The rate constants of the photodegradation reactions on MB over the as-prepared C fibers@MoSe_2_ NPCSC and commercially bought MoSe_2_ powder are 0.0043 and 0.0003 min^−1^, respectively. This result indicates that after compositing with C fibers in the form of the present C fibers@MoSe_2_ NPCSC, the rate constant of MB photodegradation over MoSe_2_ nanoplates under SSI was increased in a factor of about 14.

Fig. 8c illustrates the photocatalytic activity of the sample stored for 4 months on degrading MB under SSI. It is seen that the totally decolourized MB by the catalyst stored for 4 months was 57.3%, almost equaling to that by the fresh one (54.9%). This result reveals an excellent structural stability of the C fibers@MoSe_2_ NPCSC photocatalyst for a long period of storage.

Moreover, the sample was also repeatedly used for the photodegradation of MB to further evaluate its chemical stability. The result is displayed in Fig. 8d. It was revealed that during the repeated use, the photocatalytic activity of the catalyst for the degradation of MB under SSI decreased very little after 3 times of experiments were carried out. This result in combination of their well-kept morphology and composition as shown in the SEM images in Fig. 9 after being used indicates that such catalyst has a relatively high stability during photocatalytic application. As for the very little reduction in photocatalytic activity during recycling use, it might be resulted from the exfoliation and loss of a few MoSe_2_ nanoplates from the sample during the repeated washing and drying after each cycle of photocatalytic test.

In combination with its original morphology and composition, Fig. 9 reveals that the morphology and composition of the catalyst can be well maintained during the photocatalytic degradation of MB, indicating that the catalyst has good stability during such reactions.

It can be seen from Fig. 10a that in the dark adsorption stage, the decolourized RhB by the C fibers@MoSe_2_ NPCSC was 11.2%, while that by the commercially available MoSe_2_ powder was only 1.4%. In the photocatalytic stage, the degraded RhB over the C fibers@MoSe_2_ NPCSC reached 18.9%, but that over the commercially bought MoSe_2_ powder was only 2.8%. This result reveals that the commercially available MoSe_2_ powder has no photocatalytic activity for the degradation of RhB. However, the photocatalytic activity of MoSe_2_ nanoplates can be greatly enhanced after compositing with C fiber in the form of the reported C fibers@MoSe_2_ NPCSC. On the basis of the recorded data on the photocatalytic degradation reactions, straight lines can be fitted for the plots of *-ln(C/C*_0_*) versus irradiation time*, and the results are shown in Fig. 10b. From the fitted graph, the rate constants of the photodegradation reaction on Rhb over the as-prepared C fibers@MoSe_2_ NPCSC and commercially available MoSe_2_ powder were calculated as 0.00347 and 0.00043 min^−1^, respectively. It is seen that after composting with C fibers, the photodegradation rate of RhB over the present C fibers@MoSe_2_ NPCSC was 8 times higher than that over the commercially available MoSe_2_ powder.

Fig. 10c reveals the stability of the C fibers@MoSe_2_ NPCSC on degrading RhB under SSI. While the other conditions were fixed, the decolourized RhB by the C fibers@MoSe_2_ NPCSC catalyst stored for 4 months reached 72.6%, which is very close to that over the fresh C fibers@MoSe_2_ NPCSC (69.9%). This result indicates that after being stored for a long time, the fibers@MoSe_2_ NPCSC still had good photocatalytic performance on the photodegradation of RhB. Fig. 10d displays the photocatalytic repeatability of the C fibers@MoSe_2_ NPCSC on degrading MB under SSI. Three repeated tests were performed on the photodegradation of RhB over the same catalyst sample. It is seen from this graph that, the photocatalytic activity of the C fibers@MoSe_2_ NPCSC on the degradation of RhB decreased very little after each test. In combination with their good morphology and well-kept composition after photodegradation test as shown in Fig. 11, it was revealed that the present C fibers@MoSe_2_ NPCSC had excellent photocatalytic stability.

In combination with its original morphology and composition, Fig. 11 reveals that the catalyst could maintain its morphology and composition during the photocatalytic degradation of RhB, indicating that the C fibers@MoSe_2_ NPCSC catalyst has good stability during such reactions.

In combination with its original morphology and composition, Fig. 13 reveals that the catalyst could maintain its morphology and composition during the photocatalytic degradation of 4-CP, indicating that the C fibers@MoSe_2_ NPCSC catalyst has good stability during such photocatalytic reactions.

In combination with its original morphology and composition, Fig. 14 indicates that the morphology and composition of the catalyst can be well maintained during the photocatalytic degradation of Cr(VI), indicating that the catalyst has good stability during such photocatalytic reactions.

## References

[1] Wang M., Peng Z.J., Qian J.W., Li H., Zhao Z.Y., Fu X.L. (2018). Highly efficient solar-driven photocatalytic degradation on environmental pollutants over a novel C fibers@MoSe_2_ nanoplates core-shell composite. J. Hazard. Mater..

[2] Deurbergue A., Oberlin A. (1991). Stabilization and carbonization of pan-based carbon fibers as related to mechanical properties. Carbon.

[3] Shen Z.G., Zhao Z.Y., Qian J.W., Peng Z.J., Fu X.L. (2016). Synthesis of WO_3−x_ nanomaterials with controlled morphology and composition for highly efficient photocatalysis. J. Mater. Res..

[4] Qian J.W., Zhao Z.Y., Shen Z.G., Zhang G.L., Peng Z.J., Fu X.L. (2015). A large scale of CuS nano-networks: catalyst-free morphologically controllable growth and their application as efficient photocatalysts. J. Mater. Res..

[5] Ho W.K., Yu J.C., Lin J., Yu J.G., Li P.S. (2004). Preparation and photocatalytic behavior of MoS_2_ and WS_2_ nanocluster sensitized TiO_2_. Langmuir.

[6] Cheng Y.P., Sun H.Q., Jin W.Q., Xu N.P. (2007). Photocatalytic degradation of 4-chlorophenol with combustion synthesized TiO_2_ under visible light irradiation. Chem. Eng. J..

[7] Li X.H., Zhang H.D., Zheng X.X., Yin Z.Y., Wei L. (2011). Visible light responsive N-F-codoped TiO_2_ photocatalysts for the degradation of 4-chlorophenol. J. Environ. Sci..

[8] Yu H., Zheng X.X., Yin Z.Y., Tao F., Fang B.B., Hou K.S. (2007). Preparation of nitrogen-doped TiO_2_ nanoparticle catalyst and its catalytic activity under visible light. Chin. J. Chem. Eng..

[9] Ren Z.X., Liu X.J., Chu H.P., Yu H.Z., Xu Y.Y., Zheng W., Lei W.Y., Chen P.B., Li J.W., Li C. (2017). Carbon quantum dots decorated MoSe_2_ photocatalyst for Cr(VI) reduction in the UV–vis-NIR photon energy range. J. Colloid Interface Sci..

[10] Chu H.P., Liu X.J., Liu B.B., Zhu G., Lei W.Y., Du H.G., Liu J.Y., Li J.W., Li C., Sun C.Q. (2016). Hexagonal 2H-MoSe_2_ broad spectrum active photocatalyst for Cr(VI) reduction. Sci. Rep..

[11] Chu H.P., Lei W.Y., Liu X.J., Li J.L., Zheng W., Zhu G., Li C., Pan L.K., Sun C.Q. (2016). Synergetic effect of TiO_2_ as co-catalyst for enhanced visible light photocatalytic reduction of Cr(VI) on MoSe_2_. Appl. Catal. A-Gen..

[12] Chu H.P., Lei W.Y., Liu X.J., Qu J.H., Li J.L., Zhu G., Niu L.Y., Pan L.K. (2016). MoSe_2_ visible-light photocatalyst for organic pollutant degradation and Cr(VI) reduction. J. Mater. Sci. -Mater. Electron.

[13] Zhang R.Y., Wan a W.C., Li D.W., Dong F., Zhou Y. (2017). Three-dimensional MoS_2_/reduced graphene oxide aerogel as a macroscopic visible-light photocatalyst. Chin. J. Catal..

[14] Weng B., Zhang X., Zhang N., Tang Z.R., Xu Y.J. (2015). Two-dimensional MoS_2_ nanosheet-coated Bi_2_S_3_ discoids: synthesis, formation mechanism, and photocatalytic application. Langmuir.

