# Supplementary material for: Data on novel C fibers@MoSe2 nanoplates core–shell composite for highly efficient solar-driven photocatalytically degrading environmental pollutants
Source: Data Brief. 2018 Feb 8;17:842–55. doi: 10.1016/j.dib.2018.01.103 (PMC5834650; doi:10.1016/j.dib.2018.01.103)
Supplement: Supplementary file 1 — Supplementary material. [file mmc1.pdf]

Conflicts of Interest Statement

DIB-D-18-00058

Manuscript title: **Data on novel C fibers@$MoSe_2$ nanoplates core-shell composite for highly efficient solar-driven photocatalytically degrading environmental pollutants**

The authors whose names are listed immediately below certify that they have NO affiliations with or involvement in any organization or entity with any financial interest (such as honoraria; educational grants; participation in speakers' bureaus; membership, employment, consultancies, stock ownership, or other equity interest; and expert testimony or patent-licensing arrangements), or non-financial interest (such as personal or professional relationships, affiliations, knowledge or beliefs) in the subject matter or materials discussed in this manuscript.

Author names:

Meng Wang **Zhijian Peng** **Jingwen Qian**

Hong Li **Zengying Zhao** **Xiuli Fu**

The authors whose names are listed immediately below report the following details of affiliation or involvement in an organization or entity with a financial or non-financial interest in the subject matter or materials discussed in this manuscript. Please specify the nature of the conflict on a separate sheet of paper if the space below is inadequate.

Author names:

This statement is signed by all the authors to indicate agreement that the above information is true and correct (*a photocopy of this form may be used if there are more than 10 authors*):

Author's name (typed)	Author's signature	Date
**Meng Wang**	Meng Wang	Jan 28, 2018
**Zhijian Peng**	Zhijian Peng	Jan 28, 2018
**Jingwen Qian**	Jingwen Qian	Jan 28, 2018
**Hong Li**	Hong Li	Jan 28, 2018
**Zengying Zhao**	Zengying Zhao	Jan 28, 2018
**Xiuli Fu**	Xiuli Fu	Jan. 28, 2018
